# Bivariate multilevel modeling of antenatal care contacts and place of delivery among reproductive-aged women in Ethiopia

**DOI:** 10.1371/journal.pone.0316795

**Published:** 2025-02-10

**Authors:** Denekew Bitew Belay, Seniat Mulat, Nigussie Adam Birhan, Ding-Geng Chen

**Affiliations:** 1 Department of Statistics, Bahir Dar University, Bahir Dar, Ethiopia; 2 Department of Statistics, University of Pretoria, Pretoria, South Africa; 3 Department of Statistics, Hawassa University, Hawassa, Ethiopia; 4 Department of Statistics, Injibara University, Injibara, Ethiopia; 5 College of Health Solutions, Arizona State University, Phoenix, Arizona, United States of America; Woldia University, ETHIOPIA

## Abstract

**Background:**

Antenatal care (ANC) contacts, along with enhanced health facilities for delivery, are essential components of maternal and child healthcare, as these significantly contribute to both mothers and their newborn child’s health. Antennal care contacts primarily help women maintain normal pregnancies by detecting pre-existing conditions and preventing complications that may arise during childbirth. This study intended to determine possible factors that affect both ANC contact and place of delivery among women in Ethiopia.

**Methods:**

The 2019 Ethiopian Mini Demographic and Health Survey data were used for this study. A total weighted sample of 3,926 women nested within 68 zones was used. The bivariate multilevel logistic regression model was utilized to assess the association between antenatal care contact and place of delivery and determinant factors among reproductive-aged women in Ethiopia.

**Results:**

In this study, 57% and 47.5% of women had no ANC contacts and home delivery respectively. Similarly, about 36.73% of women delivered at home and didn’t utilize the recommended ANC contacts. Only 32.99% of women have both health facility delivery and at least four ANC contacts during their pregnancy. Women who reside in rural areas were 0.612 and 0.352 times less likely to have ANC and health facility delivery compared to women who reside in urban areas. Whereas, the estimated odds of women with higher education levels were 3.803 and 8.406 times the estimated odds of women with no education.

**Conclusion:**

A high proportion of women are still delivering their new child at home and still don’t have at least four ANC contacts during their pregnancy. Women’s age, women education level, marital status, wealth status, sex of household head, place of residence, and region were significant predictors of antenatal care visits and place of delivery simultaneously in Ethiopia. Although the country tried to maximize these services, it still requires expansion of health facilities media campaigns, and women’s literacy to reduce maternal and newborn child mortality in Ethiopia.

## Introduction

Antenatal care (ANC) visits and improved delivery facilities are crucial for maternal and child healthcare, as they improve the health of mothers and their new-borns. Regular ANC allows healthcare providers to monitor the mother’s health, detect potential complications early, and administer necessary interventions. ANC also helps women maintain healthy pregnancies by identifying pre-existing conditions and preventing complications during delivery. Improved delivery facilities ensure safe care during childbirth, reducing maternal and infant mortality risk. Place of delivery (PD) refers to the location of childbirth, either at home or in a health facility [1–4].

Pregnancy and childbirth in Sub-Saharan Africa are characterized by low rates of health facility births, with 99% of maternal deaths occurring in developing countries. Over 800 women die daily due to preventable complications worldwide [5]. In Africa, direct obstetric problems during delivery, such as haemorrhage, hypertension, sepsis, and obstructed labour, account for 64% of all maternal deaths [6–9].

Maternal healthcare service utilization remains a crucial measure for tracking advancements in maternal and child health outcomes. However, Sub-Saharan countries, which have the world’s highest maternal mortality rate (420 per 100,000 live births), are associated with low rates of recommended antenatal care (ANC) visits and skilled birth attendance [10,11]. Maternal mortality in Ethiopia was high, at 412 per 100,000 live births. Providing access to antenatal care (ANC) and skilled birth attendance can help prevent maternal and infant deaths [12,13].

A joint multilevel analysis of antenatal care (ANC) and place of delivery has not been previously well examined for women of reproductive age in Ethiopia at the zonal level. While the number of women who attended at least four ANC contacts and potential influencing factors have been identified, the variations at the zonal administration level have not been addressed and most of the studies have focused on a separate analysis using antenatal and delivery care utilization along with their determinant factors in Ethiopia [4,7,14,15].

Prioritizing antenatal care and the quality of delivery services, and a fair distribution of these services in the zone plays a vital role in fostering healthier communities and improving the overall quality of life for women and their children [16–19]. The joint analysis using both antenatal care visits and place of delivery and identifying the associated risk factor can improve the estimates and will help us to put deliverable recommendations at a zonal level which is the lowest administration region for better intervention. Therefore, this study intended to determine possible factors that affect both ANC visits and place of delivery jointly among reproductive-aged women at the zonal level of Ethiopia.

## Materials and methods

### Study setting and design

This study was conducted in Ethiopia, a country located in the horn of Africa and part of Sub-Saharan Africa, situated between 33° and 14° east longitude and 3° and 15° north latitude. A cross-sectional study design was employed using data from the 2019 Ethiopian Mini Demographic and Health Survey (EDHS), which was carried out by the Central Statistical Agency in collaboration with the Federal Ministry of Health and the Ethiopian Public Health Institute. Administratively, Ethiopia is divided into nine regional states and two city administrations, further subdivided into 68 zones [20].

### Data sources and study population

The dataset from the 2019 Ethiopian Mini Demographic and Health Survey (EMDHS) was used for this study. This survey was the second EMDHS and the fifth DHS conducted in Ethiopia, carried out from March 21, 2019, to June 28, 2019. We utilized the individual record (IR) dataset and extracted both the dependent and independent variables. The data set used in this study is freely available and possible to download by justifying the reason for requesting the data from the link: https://dhsprogram.com/data/available-datasets.cfm. All Ethiopian women (15–49 years old) in the reproductive age range are considered as the study’s population. Women in Ethiopia who had children within the previous five years of the survey for the most recent birth were involved in the study.

### Sample size and sampling procedure

The 2019 EMDHS was carried out among 8,663 households to ensure national representation. The survey included interviews with 8,885 women aged 15–49 years, with a 98.6% response rate. A stratified two-stage cluster sampling procedure was used to collect data from 9 regional states and 2 city administrations, with probability proportional to enumeration area (EA) size based on the 2019 primary healthcare frame and independent selection in each sample stratum. In the first stage, 305 EAs (93 urban and 212 rural) were chosen.

In the second step of selection, a set number of 30 households per cluster was chosen by an equal probability systematic sampling from the newly produced household list. Finally, a nationwide sample of 8885 eligible women was interviewed. Women who had not given birth within the five years before the survey were excluded from this study. As a result, the analytic sample for the current study consists of 3,926 women who had at least one live birth in the past five years.

### Study variables

#### Outcome variables.

In this study, two binary outcome variables were considered. These are antenatal care (ANC) and place of delivery (PD).


$$\text{ANC=} \{\begin{array}{c} yes\;=\text{ 1 , if a women attended four or more ANC 4 contacts/visits}\\ \text{no }=\text{ 0, otherwise } \end{array}$$

$$\text{Place of delivery=} \{\begin{array}{c} \text{1, if the woman gave birth at a health facility}\\ \text{0, if the woman gave birth at home } \end{array}$$

#### Independent variables.

The relevant risk factors associated with ANC visits and the place of delivery were included as individual-level variables: mother’s current age, mother’s educational level, family wealth index, religion, marital status, birth order, birth interval, number of children ever born, exposure to mass media, and community-level variables such as place of residence and region [18,21].

#### Data management and analysis

The data were extracted using STATA 18 software before being analyzed using SAS version 9.4 with PROC LOGISTIC and PROC GLIMMIX, which used the LAPLACE approximation. Descriptive statistics, such as frequencies and percentages, were utilized to summarize the individuals’ background information.

### Bivariate binary logistic regression

Bivariate binary logistic regression is an extension of univariate logistic regression when there are two correlated categorical response variables, such as ANC and place of delivery. This approach examines the relationship between the two correlated categorical dependent variables and their associated independent variables. In this study, we focused on these two correlated categorical dependent variables, each of which has two categories. Let $Y_{1}$ and $Y_{2}$ are the two dependent variables such that ANC and place of delivery respectively, and each can have one of the two values (0 or 1) as described in Table 1. The best method for measuring the relationship between categorical variables in the logistic regression model was the odds ratio [2,22].

**Table 1:** The (2 × 2) contingency table of the response variable.

		Y_2_ (Place of Delivery)		Marginal
		Y_2_ = 1	Y_2_ = 0	
Y_1_(ANC)	Y_1_ = 1	Y_11_	Y_10_	Y_1_+
	Y_1_ = 0	Y_01_	Y_00_	Y_0_+= n−Y_1_+
Marginal		Y+_1_	Y+_0_ = n−Y+_1_	Y++ = n

Table 2 shows the joint probability of the response variable and based on Table 1 and Table 2, the random variables. $Y_{11},Y_{10}Y_{01}$ and $Y_{00}$ follows the multinomial distribution with a joint probability function defined by

**Table 2:** The joint probability of the response variables.

		Y_2_ (Place of Delivery)		Marginal
		Y_2_ = 1 (health)	Y_2_ = 0 (home)	
Y_1_ (ANC)	Y_1_ = 1(yes)	P_11_ = p (y_1_ = 1, y_2_ = 1)	P_10_ = p (y_1_ = 1, y_2_ = 0)	P_1+_
	Y_1_ = 0 (no)	P_01_ = p (y_1_ = 0, y_2_ = 1)	P_00_ = p (y_1_ = 0, y_2_ = 0)	P_0+_= 1−P_1_ +
Marginal		Y+_1_	P+_0_ = 1−P+_1_	P_++_ =1


$$P\left(Y_{11}=y_{11},Y_{10}=y_{10},Y_{01}=y_{01},Y_{00}=y_{00}\right)=\overset{1}{\underset{0}{\prod }}\overset{1}{\underset{0}{\prod }}\frac{{\text{p}}_{\text{gh}}^{{\text{y}}_{\text{gh}}}}{{\text{y}}_{\text{gh}}},0<{\text{p}}_{\text{gh }}<1$$

where: $g,h=0,1;y_{gh}=0,1;Y_{00}=1-y_{11}-Y_{10}-Y_{01};p_{00}=1-p_{11}-p_{10}-p_{01}$.

Denote that $\psi \left(x\right)$ The odds ratio shows an association between $Y_{1}$ and $Y_{2}$ depend on *x* which shows that $Y_{1}$ and $Y_{2}$ are correlated. The variable $Y_{1}$ and $Y_{2}$ are independent if $\psi \left(x\right)=1$ [23]. The odds ratio is defined by


$$\psi \left(x\right)=\frac{p_{11}\left(x\right)p_{00}\left(x\right)}{p_{10}\left(x\right)p_{01}\left(x\right)},\psi \left(x\right)\geq 0.$$

### Multilevel bivariate logistic regression

Separate multilevel analyses of ANC and place of delivery among reproductive-age women have been conducted in various research papers [15,17,24]. However, implementing a separate analysis would ignore the dependency between the ANC and the place of delivery. To take under consideration the correlation between the ANC and place of delivery and the estimates of effects of one or more covariates, the multilevel bivariate logistic regression model is a more plausible alternative. Multilevel bivariate binary logit models were used to account for the hierarchical nature of the data that affect the outcome variable and how the interactions among covariates measured at different levels (zonal administrative level) affect the outcome variable [23,25]. As the EMDHS 2019 data was collected from women living in different zones in Ethiopia, the likelihood of having a clustering effect is very high. In this study, we considered a two-level hierarchical analysis where women are nested within zonal administration. The clustering effect was checked using the intra-class correlation (ICC) coefficient [26]. When the logistic model is used the residual at level one (women level) is assumed to follow the standard logistic distribution and the variance. $\frac{\pi^{2}}{3}=3.29$ was assumed. The ICC can be calculated as:

$ICC=\frac{\sigma_{\mu 0}^{2}}{\sigma_{\mu 0}^{2}+\frac{\pi^{2}}{3}}$, where $\sigma_{\mu 0}^{2}$ is the estimated cluster variance (zonal level).

The multilevel model includes both fixed and random effects terms. The results of the fixed effects of the model were presented as an adjusted odds ratio (AOR) while the random effects were assessed with an intra-class correlation coefficient (ICC). In this study, four models were fitted with the null model (Model 0), which shows the variations of place of delivery and ANC in the absence of any independent variables, Model I as an adjusted for the individual-level variables, Model II as adjusted for the community-level variables and model III as adjusted for both individual and community-level variables. Akaike’s information criterion (AIC) and the Bayesian information criterion (BIC) determine the multilevel model that fits the data well and select the final model. Finally, adjusted odds ratios (AORs) were estimated and statistically significant predictors of ANC and place of delivery were identified at a p-value less than 0.05.

### Ethical review statement

This study is based on Demographic and health survey (DHS) data and the DHS Program maintains standards for protecting the privacy of respondents and household members in all DHS surveys. Procedures and questionnaires for standard DHS surveys have been reviewed and approved by the International Coaching Federation (ICF) Institutional Review Board (IRB). While the host country IRB ensures the survey conforms with local laws and customs, the ICF IRB ensures it adheres to the U.S. Department of Health and Human Services’ requirements for protecting human subjects (45 CFR 46). So, country-specific DHS survey protocols are reviewed by the ICF IRB and typically by an IRB in the host country and the procedures can be found in the link: https://www.dhsprogram.com/methodology/Protecting-the-Privacy-of-DHS-Survey-Respondents.cfm.

## Result

In this study, a total weighted sample of 3,926 women of reproductive age was considered. As depicted in Table 3, from the total women who participated in this study, 2238 (57%) women didn’t utilize minimum WHO-recommended ANC contacts, and 1864 (47.5%) made their delivery at home which shows a low proportion of several antenatal care visit and a high proportion of home delivery, respectively.

**Table 3:** frequency and percentage distribution of the dependent variables.

Variable	Categories	Frequency (%)
Antenatal care contacts	No	2238(57.0)
	Yes	1688(43.0)
Place of delivery	Home	1864(47.5)
	Health facility	2062(52.5)

In addition, as seen from Table 4, among women of reproductive age, the majority of respondents 1192 (30.37%) were between the ages of 25 and 29 years. The majority of participants, 2900 (73.86%) lived in rural areas. Among the reproductive-age women, 2501 (63.69%) had no media exposure; of them, approximately 1664 (74.35%) had no ANC contacts and 1447(77.62%) had home delivery.

**Table 4:** Association of socio-demographic and obstetric characteristics with ANC and place of delivery (PD), EMDHS 2019.

Variables	Categories	Weighted frequency (%)	Antenatal care contact			Place of delivery		
			No (%)	Yes (%)	$X^{2}$ p-value	Home (%)	Health facility (%)	$X^{2}$ P-value
Age	15–19	227 (5.79)	158 (7.08)	69 (4.06)	<0.001	97 (5.22)	130 (6.30)	<0.001
	20–24	769 (19.58)	421 (18.79)	348 (20.63)		295 (15.86)	474 (22.95)	
	25–29	1192 (30.37)	636 (28.40)	556 (32.97)		559 (30.00)	633 (30.70)	
	30–34	799 (20.36)	435 (19.44)	364 (21.57)		396 (21.27)	403 (19.54)	
	35–39	591 (15.04)	362 (16.17)	229 (13.55)		305 (16.39)	285 (13.83)	
	40–44	259 (6.59)	167 (7.45)	92 (5.45)		154 (8.27)	104 (5.07)	
	45–49	89 (2.27)	59 (2.65)	30 (1.76)		56 (3.00)	33 (1.61)	
Women’s education level	No education	2014 (51.30)	1361 (60.82)	653 (38.69)	<0.001	1256 (67.37)	758 (36.78)	<0.001
	Primary	1414 (36.03)	750 (33.51)	664 (36.36)		549 (29.47)	865 (41.96)	
	Secondary	345 (8.78)	94 (4.23)	250 (14.81)		46 (2.46)	299 (14.48)	
	Higher	153 (3.89)	32 (1.44)	121 (7.14)		13 (0.69)	140 (6.79)	
Sex of Household head	Female	525 (13.38)	307 (13.75)	218 (12.90)	0.885	217 (11.65)	308 (14.95)	0.003
	Male	3401 (86.62)	1930 (86.25)	1471 (87.10)		1647 (88.35)	1754 (85.05)	
Religion	Orthodox	1441 (36.69)	688 (30.74)	753 (44.59)	<0.001	560 (30.04)	881 (42.71)	<0.001
	Protestant	1082 (27.57)	659 (29.43)	423 (25.10)		600 (32.22)	482 (23.36)	
	Muslim	1340 (34.12)	838 (37.46)	502 (29.69)		654 (35.04)	686 (33.28)	
	Others	63 (1.62)	53 (2.37)	10 (0.63)		50 (2.70)	13 (0.65)	
Region	Tigray	287 (7.30)	104 (4.62)	183 (10.84)	<0.001	77 (4.10)	210 (10.19)	<0.001
	Afar	51 (1.30)	35 (1.58)	16 (0.94)		34 (1.83)	17 (0.83)	
	Amhara	839 (21.38)	413 (18.47)	426 (25.24)		353 (18.94)	486 (23.59)	
	Oromia	1519 (38.69)	902 (40.30)	617 (36.55)		811 (43.48)	708 (34.35)	
	Somali	218 (5.55)	194 (8.66)	24 (1.43)		161 (8.66)	57 (2.75)	
	Benishangul	47 (1.20)	21 (0.93)	26 91.56)		13 (0.73)	34 (1.62)	
	SNNP	787 (20.05)	518 (23.17)	269 (15.92)		395 (21.19)	392 (19.02)	
	gambela	19 (0.49)	13 (0.58)	6 (0.36)		5 (0.26)	14 (0.69)	
	Harari	11 (0.28)	7 (0.31)	4 (0.26)		3 (0.17)	8 (0.39)	
	addis adaba	127 (3.22)	23 (1.03)	104 (6.13)		7 (0.35)	120 (5.82)	
	dire dawa	21 (0.54)	8 (0.36)	13 (0.77)		5 (0.29)	16 (0.76)	
Place of Residence	Urban	1026 (26.14)	423 (18.93)	603 (35.70)	<0.001	274 (14.71)	752 (36.48)	<0.001
	Rural	2900 (73.86)	1815 (81.07)	1085 (64.30)		1590 (85.29)	1310 (63.52)	
Children ever born	1	825 (21.02)	400 (17.87)	425 (25.19)	<0.001	209 (11.22)	616 (29.87)	<0.001
	2–4	1737 (44.25)	936 (41.83)	801 (47.45)		805 (43.19)	932 (45.20)	
	5 and above	1364 (34.73)	902 (40.29)	462 (27.36)		850 (45.58)	514 (2.93)	
Birth order	First	825 (21.02)	400 (17.87)	425 (25.19)	<0.001	209 (11.22)	616 (29.87)	<0.001
	2–3	1276 (32.50)	665 (29.72)	611 (36.19)		540 (28.99)	736 (35.68)	
	4–5	849 (21.63)	497 (22.20)	352 (20.87)		489 (26.24)	360 (17.46)	
	6 and above	976 (24.86)	676 (30.21)	300 (17.75)		625 (33.55)	351 (17.00)	
Media exposure	No	2501 (63.69)	1664 (74.35)	837 (49.56)	<0.001	1447 (77.62)	1054 (51.10)	<0.001
	Yes	1425 (36.31)	574 (25.65)	851 (50.44)		417 (22.38)	1008 (48.90)	
Wealth status	Poor	1647 (41.95)	1172 (52.36)	475 (28.14)	<0.001	1102 (59.14)	545 (26.41)	<0.001
	Middle	761 (19.39)	464 (20.74)	297 (17.60)		400 (21.46)	361 (17.52)	
	Rich	1518 (38.66)	602 (26.89)	916 (54.26)		362 (19.40)	1156 (56.07)	
Marital status	Married	3657 (93.14)	2058 (91.97)	1599 (94.69)	0.444	1739 (93.28)	1918 (93.02)	0.060
	Other	269 (6.86)	180 (8.03)	89 (5.31)		125 (6.72)	144 (6.98)	

Table 5 shows the joint and marginal probability of ANC contacts and place of delivery for pregnant women and the odds ratio between ANC contact and place of delivery was 5.435 (OR =  5.435, 95% CI: 4.72–6.25), indicating a statistically significant association between ANC contacts and place of delivery. Additionally, the results showed that 422 women (10.8%) never had ANC contacts during their entire pregnancy period and delivered their children at home.

**Table 5:** Joint and marginal probability of ANC contact and place of delivery (PD) for pregnant women.

		Y_1_(ANC)		Marginal place of delivery (PD)	Odds Ratio (95% CI)
		Y_1_ = 1 (Yes)	Y_1_ = 0 (No)		
Y_2_ (Place of delivery)	Y_2_ = 1 (health)	796 (0.203)	1266 (0.322)	2062 (0.525)	5.435 (4.72, 6.25)***
	Y_2_ = 0 (home)	1442 (0.367)	422 (0.108)	1864 (0.475)	
Marginal of ANC		2238 (0.570)	1688(0.430)	3926 (100)	

***p-value < 0.001.

Table 6 shows the joint frequency of factors and different combinations of ANC utilization and place of delivery. The descriptive results of this study indicate that the highest proportion of both without ANC contact and home delivery was observed among mothers in the age group of 25–29 years, with a total of 423 women (66.55%). Among different residential areas, the majority of women have no ANC contacts and made home deliveries were found among mothers residing in rural areas 1254 (69.13%). Similarly, in terms of women’s educational level, the descriptive statistics revealed that three-fourths of the respondents had not received any ANC contacts and home delivery took place 1032 (75.79%) (Table 6).

**Table 6:** Joint frequency distribution of risk factors for the different combinations of ANC contact and place of delivery (PD), EMDHS 2019.

Risk factor	Categories	No ANC and home delivery (%)	No ANC and health facility delivery (%)	ANC and home delivery (%)	ANC and health facility delivery (%)
Age	15–19	79 (50.13)	79 (49.87)	18 (25.93)	51 (74.07)
	20–24	237 (56.29)	184 (43.71)	58 (16.92)	290 (83.08)
	25–29	423 (66.55)	213 (33.45)	136 (24.47)	420 (75.53)
	30–34	280 (64.32)	155 (35.68)	116 (32.01)	248 (67.99)
	35–39	246 (68.04)	116 (31.96)	59 (25.87)	170 (74.13)
	40–44	126 (75.66)	41 (24.34)	28 (30.39)	64 (69.61)
	45–49	51 (85.39)	8 (14.61)	5 (17.45)	25 (82.55)
Women’s education level	No education	1032 (75.79)	329 (24.21)	224 (34.36)	429 (65.64)
	Primary	389 (51.89)	361 (48.11)	160 (24.10)	504 (75.90)
	Secondary	20 (21.43)	74 (78.57)	26 (10.32)	224 (89.68)
	Higher	1 (4.12)	31 (95.88)	12 (9.56)	109 (90.44)
Sex of household head	Female	183 (59.54)	124 (40.46)	34 (15.60)	184 (84.40)
	Male	1259 (65.23)	671 (34.77)	388 (26.38)	1083 (73.62)
Religion	Orthodox	405 (58.93)	283 (41.07)	155 (20.54)	598 (79.46)
	Protestant	431 (65.36)	228 (34.64)	170 (40.14)	254 (59.86)
	Muslim	561 (66.94)	227 (33.06)	92 (18.37)	409 (81.63)
	Others	45 (85.12)	8 (14.88)	5 (48.56)	5 (51.44)
Region	Tigray	50 (48.09)	54 (51.91)	27 (14.58)	156 (85.42)
	Afar	29 (82.00)	6 (18.00)	5 (32.66)	11 (67.34)
	Amhara	248 (60.11)	165 (39.89)	105 (24.55)	321 (75.45)
	Oromia	623 (69.12)	278 (30.88)	187 (30.34)	430 (69.66)
	Somali	151 (78.10)	43 (21.90)	10 (41.29)	14 (58.71)
	Benishangul	10 (48.18)	11 (51.82)	4 (14.00)	23 (86.00)
	SNNP	315 (60.84)	203 (39.16)	79 (29.58)	189 (70.42)
	Gambela	4 (30.20)	9 (69.80)	1 (15.63)	5 (90.66)
	Harari	3 (40.24)	4 (59.76)	1 (9.34)	4 (90.66)
	addis adaba	4 (17.99)	19 (82.01)	2 (2.27)	101 (97.73)
	dire dawa	4 (50.55)	4 (49.45)	1 (10.07)	12 (89.93)
Place of Residence	Urban	188 (44.40)	235 (55.60)	86 (14.29)	517 (85.71)
	Rural	1254 (69.13)	560 (30.87)	336 (30.93)	750 (69.07)
children ever born	1	167 (41.84)	233 (58.16)	42 (9.85)	383 (90.15)
	2–4	601 (64.21)	335 (35.79)	204 (25.47)	597 (74.53)
	5 and above	674 (74.72)	228 (25.28)	176 (38.08)	286 (61.92)
Birth order	First	167 (41.84)	233 (58.16)	42 (9.85)	383 (90.15)
	2–3	395 (59.38)	270 (40.62)	145 (23.80)	466 (76.20)
	4–5	371 (74.63)	126 (25.37)	118 (33.61)	234 (66.39)
	6 and above	509 (75.32)	167 (24.68)	116 (38.75)	184 (61.25)
media exposure	No	1177 (70.73)	487 (29.27)	270 (32.27)	567 (67.73)
	Yes	265 (46.22)	309 (53.78)	152 (17.84)	699 (82.16)
Wealth status	Poor	906 (77.32)	266 (22.68)	196 (41.32)	279 (58.68)
	Middle	301 (64.90)	163 (35.10)	99 (33.27)	198 (66.73)
	Rich	235 (39.03)	367 (60.97)	127 (13.83)	789 (86.17)
Marital status	Married	1337 (64.94)	721 (35.06)	402 (25.15)	1197 (74.85)
	Other	106 (58.73)	74 (41.27)	20 (22.82)	70 (77.98)

Table 7, shows the estimated intra-class correlation (ICC) for ANC and place of delivery. The estimated value of ICC for the null model for ANC contacts and place of delivery is 42%, which shows a high zonal clustering/zonal variability. This indicates that there is a clustering effect and is strongly suggestive that there is within-group variability that would benefit from a cluster effect because of the cluster. We also made a model comparison for four models as detailed and we found that model 3 was the best model for the final estimation which fits the data well (Table 7).

**Table 7:** Modal Comparison and Covariance Parameter Estimates.

Random effects (Measure of variation for ANC and PD)	Null model	Model 1	Model 2	Model 3
ICC	0.42	0.36	0.34	0.29
**Model fit statistics**				
AIC	10303.53	8701.06	9081.86	**8483.69**
BIC	10307.62	8792.99	9132.93	**8620.58**

ICC =  Intra-Class Correlation; AIC =  Akaike’s Information Criterion; BIC =  Schwarz’s Bayesian Information Criteria.

Null model: a baseline model without any determinant variable.

Model 1: is adjusted for individual-level factors.

Model 2: is adjusted for community-level factors.

Model 3: is the final model adjusted for individual- and community-level factor.

From Table 8, we found that the estimated odds of having antenatal care visits among pregnant women in age groups 25–29, 30–34, and 35–39 were 1.880, 2.143, and 2.279 times more likely to have recommended ANC contacts than women age group 15–19 respectively, considering the other variable constant. The estimated odds of pregnant women in age groups 20-24, 25–29, 30–34, and 35–39 were 1.542, 1.916, 2.362, and 2.641 were 1.542, 1.916, 2.362 and 2.641 times more likely than women age groups 15-19 years, respectively. The estimated odds of minimum recommended ANC visits and health facility delivery for married women were 1.452 and 1.508 times more likely than unmarried women respectively. The estimated odds of women who attained primary, secondary, and higher levels of education were 1.763, 2.823, and 3.803 times more likely than women who did not have formal education respectively. The estimated odds of health facility delivery for women who attained primary, secondary, and higher levels of education were 2.095, 4.379, and 8.406 times more likely than women who did not attain education.

**Table 8:** The estimated coefficient of the risk factor and its 95% CI for the outcome variables antenatal care utilization and place of delivery using the bivariate multilevel binary logistic model.

Covariates	Antenatal care		Pace of delivery	
	β (S.E)	OR (95% of CI)	β (S.E)	OR (95% of CI)
Intercept	1.338 (0.374)	3.811 (1.802,8.058)***	−1.931 (0.429)	0.145 (0.0614,0.342)***
**Woman’s age**				
15–19		Ref.		
20–24	0.336 (0.178)	1.399 (0.986,1.985)	0.433 (0.186)	1.542 (1.0705,2.221) *
25–29	0.631 (0.186)	1.880 (1.305,2.709)***	0.650 (0.197)	1.916 (1.303,2.819) **
30–34	0.762 (0.205)	2.143 (1.432,3.209) ***	0.859 (0.218)	2.362 (1.538,3.626) ***
35–39	0.824 (0.222)	2.279 (1.474,3.522) ***	0.971 (0.234)	2.641 (1.668,4.181) ***
40–44	0.644 (0.255)	1.905 (1.155,3.140) *	0.883 (0.266)	2.418 (1.434,4.077) **
45–49	0.486 (0.333)	1.626 (0.846,3.128)	0.644 (0.340)	1.904 (0.977,3.708)
Marital status				
Unmarried		Ref.		
Married	0.373 (0.149)	1.452 (1.080,1.953) *	0.411 (0.160)	1.508 (1.0981,2.069) *
Religion				
Muslim		Ref.		
Orthodox	0.202 (0.128)	1.224 (0.951,1.576)	−0.472 (0.002)	0.624 (0.461,0.844) **
Protestant	0.098 (0.149)	1.103 (0.822,1.481)	−0.619 (0.0002)	0.539 (0.389,0.744) ***
Others	−0.726 (0.376)	0.484 (0.230,1.018)	−0.495 (0.111)	0.610 (0.331,1.123)
Women’s educational level				
no education		Ref.		
Primary	0.567 (0.093)	1.763 (2.553,5.666) ***	0.740 (0.098)	2.095 (4.176,16.920) ***
Secondary	1.038 (0.152)	2.823 (1.467,2.119) ***	1.478 (0.191)	4.379 (1.729,2.539) ***
Higher	1.336 (0.203)	3.803 (2.093,3.807) ***	2.129 (0.355)	8.406 (3.005,6.381) ***
**Wealth status**				
Poor		Ref.		
Middle	0.227 (0.113)	1.255 (1.005,1.568) *	0.392 (0.113)	1.480 (1.185,1.848) ***
Rich	0.594 (0.112)	1.810 (1.452,2.258) ***	1.220 (0.119)	3.388 (2.682,4.279) ***
**Sex of household head**				
Female		Ref.		
Male	−0.236 (0.108)	0.790 (0.637,0.980) *	−0.445 (0.121)	0.641 (0.505,0.814) ***
**parity**				
1		Ref.		
2-4	−0.378 (0.221)	0.686 (0.444,1.059)	−0.937 (0.235)	0.392 (0.247,0.623) ***
5 and above	−0.262 (0.167)	0.770 (0.553,1.070)	−1.031 (0.182)	0.357 (0.249,0.510) ***
**media exposure**				
No		Ref.		
Yes	0.142 (0.094)	1.152 (0.956, 1.389)	0.024 (0.103)	1.024 (0.835,1.255)
**Birth order**				
First				
2–3	0.216 (0.198)	1.241 (0.912,1.986)	0.216 (0.206)	1.241 (0.828,1.860)
4–5	0.041 (0.143)	1.042 (0.956,1.677)	0.041 (0.146)	1.042 (0.782,1.389)
6 and above	0.301 (0.097)	1.352 (1.075,1.579)	−1.105 (0.104)	0.331 (0.972,1.464)
**Community variable**				
place of residence				
Urban		Ref.		
Rural	−0.491 (0.116)	0.612 (0.485,0.772) ***	−1.044 (0.137)	0.352 (0.267,0.463) ***
Region				
Tigray		Ref.		
Afar	−1.033 (0.299)	0.356 (0.098,1.286)	−2.093 (0.320)	0.123 (0.0311,0.489)
Amhara	−0.376 (0.281)	0.687 (0.205,2.300)	−0.484 (0.290)	0.616 (0.177,2.143)
Benishangul	−0.255 (0.363)	0.775 (0.162,3.699)	−0.041 (0.374)	0.960 (0.192,4.804)
Gambela	−1.758 (0.295)	0.172 (0.048,0.614) *	−0.943 (0.302)	0.389 (0.106,1.429)
Harari	−1.330 (0.515)	0.264 (0.029,2.429)	−1.081 (0.532)	0.339 (0.034,3.340)
Oromia	−0.653 (0.286)	0.520 (0.152,1.785)	−1.134 (0.299)	0.322 (0.089,1.168)
Snnpr	−1.067 (0.297)	0.344 (0.096,1.232)	−0.678 (0.306)	0.508 (0.136,1.892)
Somali	−2.566 (0.372)	0.077 (0.016,0.380) *	−2.414 (0.360)	0.089 (0.019,0.421) *
Addis Abeba	−0.175 (0.324)	0.840 (0.209,3.378)	−0.426 (0.424)	0.653 (0.106,4.045)
Dire dawa	−0.303 (0.517)	0.739 (0.080,6.816)	−0.805 (0.534)	0.447 (0.045,4.443)
Odds Ratio		6.381 (5.518, 7.81)		P-value < 0.0001

*Key: S.E: standard error; Ref: Reference group; p-value < 0.01 * : p-value <  0.001**: p-value <  0.0001 ***.

The estimated odds of women with middle and rich wealth status were 1.255 and 1.810 times more likely to have a minimum recommended ANC contact as compared with poor economic status women, respectively.

The estimated odds of women who live in rural areas are 0.612 and 0.352 for ANC utilization and place of delivery and this shows that the odds of minimum recommended ANC utilization and facility utilization are 0.612 and 0.352 times less likely than women who live in urban areas.

The odds of women for facility delivery who lived in male-headed households were 0.641 and this indicates that the likelihood of having facility-based delivery is 0.641 times (AOR = 0.641; 95% CI: 0.505, 0.814) less likely to deliver in a health facility than women in female-headed households.

The estimated odds of place of delivery among women who parity of 2-4 and 5 and above were 0.392 and 0.357 and this indicates that the odds of facility delivery is 0.392 times (AOR =  0.392; 95% CI: 0.247–0.623) and 0.357 times (AOR = 0.357; 95% CI: 0.249–0.510) less likely to deliver institutionally compared to women who had one child. The estimated odds ratio between ANC utilization and place of delivery was 6.381 [OR =  6.381, 95% CI: (5.518, 7.81)] indicating that there is a statistically significant association between the two outcome variables (Table 8).

## Discussions

This study aims to examine the association between minimum recommended ANC visits and place of delivery among reproductive-age women based on the bivariate multilevel logistic models in Ethiopia using the 2019 EMDHS. The model is designed to evaluate the dependency between minimum recommended ANC visits and place of delivery as well as to estimate the clustering effect given other covariates. From this study, we found that 57% of reproductive-aged women have no recommended ANC, and 47.5% of births were attended at home.

The mother’s age, mother’s marital status, mother’s education level, family wealth index, sex of household head, and place of residence are important determinant factors that have a significant effect on recommended ANC utilization. The mother’s age, mother’s marital status, mother’s religion, child’s born, mother’s education level, wealth index, sex of household head, and place of residence are important determinant factors that have a significant effect on place of delivery.

The current study showed that the age of mothers was a major factor in antenatal care (ANC) utilization. As the age of the mother rises, the likelihood of receiving antenatal care (ANC) Services also increases. This finding supports previous studies done in different countries [27–29] that showed the positive association between ANC contacts and increased age of women. This might be because health conditions and birth complications are higher in older women who tend to demand more contact [30].

This study also revealed that the education level of mothers was significantly associated with recommended antenatal care contacts and it shows that the odds of attending ANC visits among mothers who attended with primary, secondary, and higher educational levels were 1.763, 2.823, and 3.803 times the odds that women with no formal education. This finding is consistent with the studies conducted in Pakistan [30], East African countries [31] and Ethiopia [32]. This could be explained by the fact that mothers with higher levels of education are more likely to use antenatal care, have a better understanding of information, and are more aware of the necessity of the service [33]. Furthermore, educated women are more likely to improve their independence, self-confidence, and ability to make health-related decisions for themselves.

The wealth index of the household was found to be significantly associated with ANC utilization in this study. The study shows that mothers who reside in middle and rich households were 25.5% and 81.0% more likely to utilize recommended ANC compared to mothers who reside in poor households’ income, respectively. This result is consistent with the findings in Georgia [34], India [35] and Ghana [36]. This might be due to the difficulty that women in poor households face with out-of-pocket expenditure associated with the service. Women are responsible for transportation and other indirect cost to receive the service, even though the service is offered free of charge [37]. In this context, wealthier women can manage transportation and work commitments, facilitating timely ANC contacts. In contrast, women with low economic status might not attend ANC due to fear of unexpected payments and prioritizing daily expenses over their health [38].

In this study marital status affects ANC utilization and married women were more likely to attend ANC compared with their unmarried counterparts consistent with a study conducted in Ghana [39]. This could be due to the psychosocial and financial support received from their husbands, planning/desirability of their pregnancy, and the societal acceptability and support of their pregnant state when compared with their unmarried counterparts [40,41]

Place of residence was another factor for the utilization of recommended ANC in Ethiopia. The study showed that women who lived in rural areas were less likely to get recommended ANC than urban resident women [AOR =  0.612, 95% CI =  0.485–0.772].

The place of residence was significantly associated with the usage of antenatal care services. This shows that women who were living in rural areas were less likely to receive recommended ANC services than those living in urban areas. This finding is consistent with the study conducted in Nigeria [42] and Kenya [43]. This might be because the health infrastructures in the rural area are less developed and there are fewer trained health workers to give information and education about recommended ANC. In addition, place of residence also significantly affects place of delivery in which rural women were 64.8% less likely to deliver at a health facility compared to the odds that urban women deliver at a health facility. This finding was supported by studies conducted in Kenya [44], Guinea [45], Pakistan [46], and Ethiopia [47]. The possible justification might be women in an urban area easily get access to health knowledge, have financial accessing institutional/skilled personnel assistance, and have proximity to health facilities.

This study showed that middle and high-wealth status increased the likelihood of institutional delivery. This is consistent with previous studies conducted in Ethiopia [48], and Bangladesh [49]. This might be because the economic capability of the households and costs related to transport might influence the preference of the place of delivery. Besides, mothers from higher wealth status might be more likely to utilize maternal health services compared to others.

Moreover, women with a higher educational status had a higher likelihood of institutional delivery than women who have had no formal education and the finding is consistent with previous studies conducted in Ethiopia [50,51] and Tanzania [52]. This study showed that mothers who reside in middle and rich wealth status households increased the likelihood of health facility delivery compared to mothers who reside in poor households. This is consistent with previous studies conducted in Ethiopia [48,53], Bangladesh [49] and Pakistan [46]. It could be reasoned that the economic capability of households and the costs related to transport might influence the preference for health facility deliveries. Besides, these mothers from better economic status might be pursuing maternal health services and the capacity to make decisions about deliveries from health facilities than others [54]. In contrast, financial problem leads to poor maternal health care.

The findings of this study also showed that the educational level of mothers significantly affects the place of women’s delivery. It revealed that women who attained primary, secondary, and higher education had a higher likelihood of health facilities delivery than women who had no education. This finding is consistent with previous studies in Ethiopia [16,50,51], and Tanzania [52]. This might be due to the optimistic attitude of educated women that might enhance women’s self-determination; and the knowledge of the drawbacks of home delivery. The finding of this study also indicated that the mother’s age is an important determinant of the place of deliveries. The likelihood of institutional delivery increased as women’s ages increased beyond the age range of 15–19 years, which is consistent with a study in Ethiopia [55]. The reason might be due to the young women’s fear of complications during home delivery, and it might be also due to maturity and understanding of the safety and other benefits of giving birth in healthcare facilities. As age increases, there will be increased knowledge and ability to make beneficial decisions regarding maternal health services.

Furthermore, mothers who live in the Somalia region had lower odds of health facility delivery compared to the reference category (Tigray region), and this result is consistent with other studies in Ethiopia [50,56]. The possible explanation might be the inaccessibility of health facilities in Somalia regions and people might have difficulty having permanent residency access to the services. The health facility delivery decreased with a high parity of women, which is consistent with the study conducted in Bangladesh [49], Ghana [57], and Pakistan [58]. This might be the service quality given in previous births. Even if the Ethiopian health system has improved in the previous decade, still there were critical shortages of health personnel, supplies of drugs, and equipment. This could discourage women from utilizing health services in later pregnancies for delivery. Similarly, Women who lived in male-headed households were less likely to deliver in a health facility than women in female-headed households. The findings of this study are in agreement with those of a study conducted in Tanzania[59].

## Strengths and limitations of the study

The strengths of the study are data from a nationally representative population-based study with appropriate weighting, as well as useful, high-quality data on mothers, households, and communities. Furthermore, the study has a large sample size drawn at random across the country, allowing results to be generalized to women of reproductive age. Furthermore, when using binary logistic regression, the relationship between ANC visits and the place of delivery is ignored. The researcher employs a model that accounts for the data’s hierarchical structure. This statistical model is used to simultaneously simulate two binary outcome variables and assess their relationship to other predictors. The major limitation of this study was a cross-sectional survey, which may not help establish a temporal relationship between the possible risk factors of pregnant women’s ANC visits and place of delivery. Moreover, the data was self-reported; there might also be a possibility of recall and social desirability biases that will result in underreporting and misreporting of events.

## Conclusion

This study aims to examine the association between ANC contacts and place of delivery among reproductive-age women based on the bivariate multilevel logistic models in Ethiopia using the 2019 Ethiopian min demographic and health survey data. The model applied in this study is designed to evaluate the effects of risk factors on ANC and place of delivery as well as to estimate the clustering effect. In this study, out of 3926 reproductive-age women, 57% of women had below the minimum recommended ANC contacts and 47.5% of the total women made their delivery at home. Of the total women considered in the study, about 36.7% of them delivered their new child at home and they didn’t utilize the recommended ANC during their pregnancy period. Only 32.2% of the women have made both facility-based deliveries and got the minimum WHO-recommended ANC contact during their pregnancy. Women’s age, women education level, marital status, wealth status, sex of household head, residence, and region were significant predictors of antenatal care and delivery care utilization simultaneously in Ethiopia.

It would be useful to increase financial support strategies that enable pregnant women from poor households to use health services and enhance pregnant women’s understanding of the significance of recommended ANC and institutional delivery through health education targeting women with their level of education. Emphasis should also be placed on supporting unmarried pregnant women to have recommended ANC and institutional delivery. Ministry of Health, health facility professionals, and community health workers have an important task in raising mothers’ attitudes to ANC utilization and institutional delivery.

## Supporting information

S1 File Antenatal and delivery care weighted. (DTA)

## Abbreviations

ANC: antenatal care
EMDHS: Ethiopia Mini Demographic Survey
ICC: Intra-Class Correlation
AIC: Akaike’s Information Criterion
BIC: Schwarz’s Bayesian Information Criteria
